# Repertory of the Back

**Published:** 1899-01

**Authors:** E. H. Wilsey

**Affiliations:** Parkersburg, W. Va.


### REPERTORY OF THE BACK.

E. H. Wilsey, M. D., Parkersburg, W. Va.

Continued from November No., p. 500.

### SCAPULÆ.

**Under** inferior angle of scapulæ a sticking during expiration, Physos.
— left scapula, a cutting, Thuya.
— scapulæ, a cramp-like pressure, Lyss.
— right scapula, aching at 11 A. M., then between, Merc.
— right scapula, an upward pressure, Calc-c.
— right scapula, aching when awaking, extending over hepatic region, Merc.
— both scapulæ, a soreness, Nat-ars.
— left scapula, intermittent aching during day, worse on moving, Lappa.
— left scapula, dull on percussion covering size of hand, Chelid.
— right scapula, a sharp pressure at any motion of the arms, Con.
— lower portion of left scapula, a throbbing and beating, Zinc.
— obtuse stitch under right scapula, while inspiring, Colocy.
— scapulæ, dull stitches, Asar.
— right scapula a sharp tearing stitch, Kali-c.

### SCAPULÆ.

**Under,** a cutting in scapulæ after dinner with pressure in stomach, Hepar.

— right scapula, a pain, better by motion, Bapt.

— dull pain under right scapula, *Chenop-ant.*, Indium.

— stitch-like pain under right scapula when stooping, *Jug-cin.*

— drawing a long breath, worse when under right scapula, Jug-cin.

— left scapula, a shooting pain, *Calc-c.*

— right scapula, a pain when bending forward, Lob-inf.

— right scapula and between scapulæ, a pressing pain, Lyss.

— a terrible pain under left scapula, around to chest and down to groin, constricting chest and affecting breathing, *Mag-phos.*

— right scapula a pain, Elater., Med., Podo., Sepia.

— constant pain under lower inner angle of right scapula, extending to chest or stomach, causing nausea and vomiting, *Chelid.*

— right scapula, a constant pressive pain, Nat-m.

— fixed pain under inner lower end of right scapula, with frightful cough, diarrhœa, and exhaustion, *Chelid.*

— left scapula, a pain, Ailanthus, Arundo., *Cactus.*, Hydrast., Helleb., Indium., Iod. (Gels. at 7 P. M.), Myrica at 11 P. M..

— stitches under the scapulæ, with outward pressing pain, Cepa.

— a sharp pain under left scapulæ, Arundo., Daphne.

— left scapula, a sharp pain near axilla, Lappa.

— scapulæ, a pain, worse on moving, Apis.

— dull, but quite severe pulsating pain under lower angle of left scapula in afternoon, Sinapis-nigra.

— right scapula, a pain, in back a pressive pain, which on inspiration changes into a lancinating pain, Cup.

— right scapula, a pain close beside the spine, as from needle-pricks, Aur-fol.

— pain under point of scapulæ, and between shoulders, extending into loins, Ox-ac.

— sore pain at point under left scapula, Papaya-vul.

— a heavy pain after dinner under left scapula, Physos.

### SCAPULÆ.

**Under** left scapula, a burning with pain through shoulder, *Cund.*

— pain under scapulæ, and in small of back, Elater.

— pain under scapulæ, with chill, Sang.

— pain under right scapula, as if chest would burst when coughing or drawing a long breath, Senega.

— slight pain in left side under left scapula, also in left hip, Xanthox.

— pain in left scapula and in neck, *Gels.*

— pain under both scapulæ, a painful tension in muscles when at rest, and much worse by raising arm, Con.

— pain, piercing close under left scapula, drawing for a few minutes while sitting, *Millef.*

— tensive pain under left scapula while respiring, *Kali-C.*

**Upper,** rheumatic pains upper part of left scapula, after the usual bath, Carbo-v.

— scapular region in a pressive pain with stiffness on sitting still, worse on beginning to move, Indium.

— third dorsal vertebræ, a violent pain, extending through shoulder-blades, *Kalmia.*

— part of scapulæ, a tensive pain, during motion, Caustic.

**Violent** pain in lower angle of left scapula, *Chelid.*

**Waking,** aching under right scapula on awaking, extending over hepatic region, Merc.

**Weakness** in region of scapula, better by stooping, Alum.

**Works,** there is a sticking in scapula, if she works a little with hands, Fer.

**Yawning,** sudden aching under lower angle of right scapula, passing off gradually, better by bending shoulders back, with constant yawning after the pain, Conval.

### REPERTORY OF THE LUMBAR REGION.

**Abdomen,** lumbar region sore internally, extending toward, Nat-c.

— cutting, drawing in lumbar region, beneath short ribs and in

LUMBAR REGION.

fore part of left side of lower abdomen, just above pubes, Rheum.

**Abdomen,** from left loin, transversely through abdomen after dinner, worse below umbilicus and toward right groin, Ran-b.

—sensation as of a bladder filled with air in left supra-iliac region, lying beneath the walls of the abdomen and pressing outward when lying on abdomen, better by lying on back, Bry.

— pain in lumbar region at night so he had to lie on abdomen, Nit-ac.

— sore pain extending around from loins externally, Bar-c.

**Abscess** near lumbar vertebræ, *Cal-phos.*

**Abscess, Psoas,** *Sil.*, *Cup-m.*, *Phos-ac.*, Symphytum., Arn.

— Psoas, first left, then right, latter discharged more than a quart of greenish, offensive pus, Syph.

— constriction of psoas muscle after abscess, *Lach.*

— impending in psoas muscle, *Asaf.*

**Aching** in general in lumbar region, *Æscul-h.*, *Actea-r.*, Apoc., Ars-h., Amyl-n., Bapt., Brach., *Bryonia.*, Calc-fl., Carbo-a., Carbo-v., Carbolic-ac., *China.*, *Cochi.*, *Cinnab.*, *Gels.*, *Helon.*, *Hydrast.*, *Hyper.*, Ind., Jugl., Kob., Lact-ac., Lyc-vir, Lyss., Med., Myr-cer., *Nux.*, *Phos.*, *Pic-acid*, *Podo.*, Ptelia, *Rhus*, *Sepia*, *Sil.*, Tel., Ustil.

— dull in lumbar region and sacrum; cannot walk; the muscles will not obey the will, *Gels.*

— across lumbar region, feels tired and weak, *Helon.*

— dull aching pain in lumbar region and back, Indium.

— or shooting pains in lumbar region, Jug-c.

— dull in lumbar muscles, Bry.

— in left lumbar region, then in both, Calend.

— in os sacrum and lumbar region, worse by stretching and bending backward, especially by pressure, Fluor-ac.

— around loins from sitting, and when arising at night, Pallad.

— persistent intermittent aching in lumbar region day and night, no better from any position, with severe sore throat, *Phyto.*

LUMBAR REGION.

**Aching** and dragging in lumbar region, worse on motion, *Pic-ac.*
— and tired feeling in lumbar region on awaking, Pic-ac.
— across lumbar region, with stiffness, Onos.
— lumbar pains and distress, soreness on waking, or awakes him at 4 A. M., Puls.
— pain in lumbar region, *Lach.*
— in lumbar region, intermittent, sharp, with constant distress, Lept.
— severe in lumbar region, continuous, Lyco-vir.
— flying pains in muscles, with persistent aching in loins and occiput, worse on movement, Lyco-vir.
— across loins, *Chin.*
— in lumbar region following seminal emissions with weakness of legs, Cobalt.
— and coldness in lumbar region and legs, *Carbo-an.*
— a rheumatic drawing and aching from loins to coccyx, *Carbo-veg.*
— dull in lumbar region, *Bapt.*
— violent in lumbar region, | Cimic.
— and dull pain, better by rest and worse by motion, Cimic.
— in cervical, lumbar, and sacral region, worse by motion, Æsc.
— dull pain in lumbar region, worse by motion, Æscul., Hydrast.
— intolerable in lumbar and sacral region, Variol.
— in loins, shooting down legs, *Sil.*
— tired over crest of ilium and worse in right side down into pelvis with general languor and desire to lie down, Spig.
— in lumbar region for three months, worse from movement (cerebro-spinal pain), *Ox-ac.*
— in lumbar region with ovarian tumor, *Apis.*
— in lumbar region when rising, *Calc-c.*
— in lumbo-sacral region, *Æsculus-hip.*, Sil.
— in lumbar region, severe in ague, *Podo.*
— in lumbar region in small-pox, *Hydrast.*
— and sore pain across lumbar region, *Lac-ac.*

### LUMBAR REGION.

**Aching** in lumbar region on stooping, Jugl.
— in lumbar region while sitting as after long stooping or bending, *Rhus.*
— pain across loins, worse on moving, *Sep.*
— dull across lumbar region when going to bed, Bapt.
— dull aching and worse from walking, Bapt.
— across lumbar region from fatigue, Amyl.
— in lumbar region with fever in the P. M., Tromb.
— in lumbar region with fistula, *Lach.*
— in lumbar region with pain in the head (menorrhagia), *Coccus.*
— in lumbar region, heavy, extending down the buttocks, *Kali-c.*
— in lumbar region with irritable bladder, Sepia.
— in lumbar region with labor-like pains on fifth day after premature labor, Lac-can.
— in lumbar region with laughter, *Cann-i.*
— in lumbar region, severe in amenorrhœa, *Lach.*
— in lumbar region with mental disorder, *Lach.*
— in lumbar region, like toothache, with a feeling of weight when she stands, which makes her sick and good for nothing, *Colch.*
— violently in lumbar region with uterine affection, *Actea-rac.*
— in lumbar region after washing (diarrhoea, prolapsus uteri), *Pod.*
— in lumbar region with feeling of languor, Zinc.
— right lumbar region to back a cutting, *Chel.*
— loin, a pain as if broken, with loss of sexual power, *Cocc-c.*
— suffers much from pain across lumbar region, *Arg-m.*
— loins, a sense of great weight, *Arn.*
— sensation as if a cord was drawn tightly across lower loin, drawing down and also in, *Arn.*
— pain across loins with coldness of extremities, *Kali-bi.*
— backache across lumbar region, *Helon.*
— gurgling in the left side of the lumbar region extending across it, Lyco.
— aching across lumbar region with stiffness, Onos.

### LUMBAR REGION.

**Aching,** lumbar region, a pain, Cund.
— dull throbbing pain in left lumbar region, worse by lying down, was compelled to walk around room with walking-stick pressed across the back to obtain relief, Vib-op.
— aching across loins, *Chin.*
— an aching pain across loins, worse on moving, *Sepia.*
— pains across loins passing to hips, *Nux-v.*
**Acute** pain in right loin in morning, Stann.
— pain P. M. and evening worse in region of lumbar vertebræ, Glon.
— drawing pain in lumbar region, worse by moving body with increase of urine, Nit.
**Action,** intense, of lumbar and dorsal muscles in opisthotonos, Oenanth.
**Agony,** excruciating in lower part of the back extending down thighs, Ox-a.
**Alternating** stitches above the right hip, Carbo-a.
— lumbago with headache, Aloe.
**Anxiety** in lumbar region after menses, Bar-c.
**Apoplexy,** pain in lumbar region followed by apoplexy and paralysis, *Bar-c.*
**Articulation,** pain in the articulation of the lumbar vertebræ and sacrum, left side pressing on spot causes pain down thigh to knee, Nat-phos.
**Automatically,** loss of sensation in muscles of lumbar region in forenoon while walking so muscles acted, Bry.
**Band,** pain in lumbar region after eating as from a band just above hips, Cina.
— pain in both loins as if surrounded by bands, Caustic.
**Bandaged,** pressure above the loins, with sensation in legs as they were stiff and bandaged, Nat-m.
**Bearing** down in lumbar region, Onos.
**Beaten** pain as if weak or beaten in lumbar region as after child-birth, during day, Nux-v.
— pain in lumbar and sacral region as if beaten, Bry.

### LUMBAR REGION.

**Bed**, jerking in lumbar region in bed in evening, Sul.
— lumbar pains of three weeks' duration after a strain ; great pain and soreness after getting warm in bed and on beginning to move, Rhus.
— a sprained pain over the hips in the P. M. and evening in bed, Sep.
— paralytic pain in loins at 10 P. M. in bed, with dullness and pain in head, Kalmia.
— gnawing in lumbar region, worse at night while in bed, Lil-tig.
**Bend**, lumbar vertebræ bend forward with curvature of spine to the left, *Calc-Phos.*
**Bending**, sore pain in lumbar region when bending over to either side, Cornus.
— dull, sharp pain in lumbar region, worse by bending spine, Diosc.
— a stiffness of muscles in lumbar region while bending over for a, short time, causing great difficulty when assuming an erect posture, *Hydrast.*
— or turning, worse pain in left lumbar region or vertebræ. Ginseng.
**Biting** and boring in lumbar region, Canth.
**Bladder**, sensation of a bladder filled with air in the left supra-iliac region, better by lying on back, Bry.
**Blow**, a pain as after a blow had been received above the hip, close by the lumbar vertebræ, Dulc.
— pain in lumbar muscles as from a blow from a fist, Nux-m.
**Bones**, tearing in lower lumbar vertebræ, extending to iliac bones, Chel.
**Boring** in loins, Carbol-ac.
— and biting in lumbar vertebræ, or region, Canth.
— in left loin from within, better from inspiration, Asaf.
**Break** lumbar region feels as if it would break, caused by a draught of air, Nux-v.
**Breath**, a dull stitch outward in left loin, close above the hip at every breath, Dulc.

### LUMBAR REGION.

**Breath,** pain in loins as if pierced with red-hot irons two mornings, worse by moving, arresting the breath and nearly causing faintness, next day pain as if strained, preventing him from straightening or stooping; during the pain in the loins the urine is scanty, of a yellowish ochre color, thick with yellowish green sediment, Bufo.

— stitches in the lumbar region taking away the breath till evening, with pain in the head and nape of neck, frequent alternations of chills and heat with apprehension in pit of stomach, Sul.

— tearing pains in lumbar muscles which arrest the breath, Kali-c.

**Breathe,** tension in lumbar region so that he could not breathe easily, Nit-acid.

**Breathing,** pain in lumbar region, worse in right loin after a short walk, worse on motion, deep breathing or sitting up straight, Convall.

— sudden violent jerks in lumbar region during a walk in open air arresting the breathing, *Ran-scl.*

— sticking in lumbar region on deep breathing, Phelland.

— severe stitches in lumbar region when breathing deeply, Nat-m.

— dull stitches in right loin only when not breathing, Clematis.

— stitches just above the loins on deep breathing, exteading into the thighs with every breath, Carbo-an.

**Broken** feeling in the lumbar region, *Cham.*, *Carbo-an.*, Clem., Coral, *Ham.*, Sepia., *Kreos.*, *Lycopod.*, *Arsenic.*, *Phos.*, *Magn-c.*, Fer-iod., *Graph.*, Melil., *Cocc-c.*

— pains across loins as if broken, with loss of sexual power, *Cocc-c.*

— drawing pain in the loins when walking, standing, and lying, with a feeling as if broken, *Carbo-an.*

— pain, as if broken, in lumbar region, cannot move, *Phos.*

— violent pain on movment or coughing, from straining back by lifting, *Rhus.*

### LUMBAR REGION.

**Broken** pain in lumbar region, as if broken, with hard stool and colic, as if the intestines would break, *Lyco.*

— feeling in lumbar region at night, *Magn-c.*

— feeling in lumbar region, especially on being touched, *Graph.*

— pain in lumbar region, as if broken to pieces, in morning in bed, Staph.

**Bruised** feeling in lumbar region, Amm-m., Acon., Æsc-h., Agari., **A**lum., Arn., *Ars.*, Berb., Bary-c., *Cham.*, *Cina.*, *China*, *Bryon.*, Calad., *Calc.*, *Eup-perf.*, Clem., *Hepar.*, *Lac-c.*, Cocc-c., Nux-m., *Sil.*, Nat-sul., *Sul.*, *Rhus*, Sul-ac., *Graph.*, Grati., Hydroctyle, Rhod., *Ruta.*, *Kali-iod.*, *Meny.*, Nux-v., Thuja, Bov., Gambog., Plat., Bufo., *Kali-n.*

— or beaten in lumbar region, morning when rising, as if, *Nat-mur.*

— feeling in lumbar region, as if crushed, Berb., Amm-m.

— feeling as if knocked away in the lumbar region, Arg-m.

— feeling in the lumbar region, with thin, white leucorrhœa, *Kali-n.*

— feeling in lumbar region, with nephritis, *Kali-i.*

— feeling, with a paralytic pain on moving, in lumbar region, *Calc-c.*

— feeling, compelling a change of position during a chill, Rhus-t.

— feeling in lumbar region, better by walking, Thuja.

— feeling in lumbar region, causing limping when walking, Hepar.

— gnawing in spot in lumbar region, and, after pressure upon it, only a bruised pain, Sul.

— pain, worse by rest and in rainy weather, Rhod.

— sensation in right side of lumbar region and in small of back, Rhus.

— pain in loins, Gratiola., Hydroctyle.

— pain in lumbar vertebræ, *Ruta.*

— pain in lumbar region, worse by sitting bent, stitches, *Kali-iod.*

— pains in the loins during the menses, Bary-c., Magn-s.

### LUMBAR REGION.

**Bruised** feeling in region of loins, especially while lying or sitting, Agar.

— feeling in lumbar region when sitting, and when standing, Sul-ac.

— pain in lumbar region, extending to neck, Acon.

— pain in lumbar region, Cinnab.

— pain in lumbo-sacral region and in groins, Cocc-c.

— sensation from shoulder in morning instead of the habitual pain in small of back, Ox-ac.

**Burning** in lumbar region, *Phos-ac.*, *Helon.*, *Canth.*, *Ran-b.*, *Nit-ac.*, *Nux-v.*, *Berb.*, *Coloc.*, *Magn-m.*, *Phos.*, *Kreos.*, *Lach.*, *Tereb.*, Alum, Ars-iod., Nat-c., Rhus, Mang., Bary-c., Arundo, Clem., Cup-m., Guaraea., Zinc., Lac-can., Med., Asar., Zizia., Lac-def., Murex., Lachn.

— like fire in lumbar region, *Lach.* (amenorrhœa).

— in lumbar region during menses, Med.

— intense in lumbar region, Lac-def.

— in lumbar region during lying-in time, Cup-m.

— in lumbar region during the sixth month of pregnancy, Rhus.

— in back, with seminal emissions nearly every night, *Phos.*

— in lumbar region, intense, with frequent urination at night, *Tereb.*

— from lumbar region to between scapula, Thuja.

— in lumbar region, in typhoid abdominalis, Nux-v.

— pain in a small spot, better by rubbing in the lumbar region, Phos.

— pain in a small spot above left pelvic region toward first lumbar vertebra, Mang.

— in the loins, transversely through body, Bar-c.

— in lumbar region, with great debility and profuse night sweats, and symptoms of slow hectic fever and violent smarting, *Phos-a.*

— and itching like a flea-bite in the region of the left loin, so that he shudders, Alum.

— heat in lumbar region, as if clothes were on fire, Ars-iod.

### LUMBAR REGION.

**Burning**, cutting and scraping in lumbar region, Nat-c.
— feeling in the loins, Rhus.
— and tired aching in lumbar region and sacrum on sitting, *Helon.*
— stitches in right lumbar region when walking, with a slight burning sensation, *Ran-b.*
— paroxysmal cutting and burning pain, with sensitiveness to slightest touch, alternating with pain and burning in tip of penis, urging to urinate and painful micturition by drops of bloody urine; at times passage of pure blood with clots, *Canth.*
— in right lumbar region, disheartening, and made him unable to think or work, Nit-ac.
— in lumbar region, extending through thigh on stepping and walking, *Nux-v.*
**Calves**, dull pain in loins, going down back of legs to lower part of, *Phos-a.*
**Caries** of lumbar vertebræ, Sil.
**Chill** in lumbar region, *Eup-Pur.*, *Nux-v.*, *Lach.*, Med., Spong., *Dulc.*
— distressing pain in lumbar region before chill, *Eupat-pur.*
— throbbing pain in lumbar region during shaking chill, *Nux-v.*
— commences in lumbar region, *Lach.*
— begins in lumbar region and spreads over whole body, *Eupat-pur.*
— running up and down lumbar region, lasting about an hour, Med.
**Chilliness** in lumbar region, *Dulc.*, Kali-iod.
— general, with pain in lumbar region on going out-doors, Myrica.
— in lumbar region at 3 P. M., *Eupat-pur.*
— creeps up from lumbar region 6 to 8 P. M., *Sul.*
— with heat in stomach, Camph.
— pain worse from pressure with chilliness in lumbar region, Æsc-h.

### LUMBAR REGION.

**Chilliness** after stool in lumbar region, *Puls.*

**Church**, tired feeling in right lumbar region in evening while sitting in church, Fer-phos.

**Coition**, drawing in lumbar region, spine and thighs after coition, Nit-ac.

**Coldness** in lumbar region, *Camph.*, Spong., Canth., Cup-s.

— dull thrust with sensation of external coldness against him, Stann.

— sensation of coldness and formication in lumbar region on a spot size of the hand, Canth.

— in lumbar region spreads over body, Cup-s.

— inner in lumbar region, worse walking, *Camph.*

— and aching in lumber region and legs, *Carbo-an.*

— pain in loins as from cold or over-lifting, Valer.

— lame in loins after taking cold, *Dulc.*

**Constriction,** pain as if a band passed through lumbar region, as if everything were constricted, taking away her breath, especially in morning, *Puls.*

— around hips, ending in the pubes, *Lil-tig.*

— in lumbar region, prevents walking, Arundo.

— painful from abdomen to lumbar region, *Calc-c.*

— in lumbar region, worse after stool, Tabacum.

**Constricting** pains from short ribs to lumbar region, *Camph.*

**Contractive,** spasmodic pain in lumbar region, *Magn-m.*

**Cord,** sensation as of a cord tightly drawn across lower loins, drawing down and also in lumbar region, *Arn.*

**Cough,** inability to cough or sneeze on account of pain in lumbar region, Nux.

**Coughing,** violent pressive pains in lumbar region, worse when at rest, so that she has to walk about to alleviate it; by gently stroking the pain is better, but by coughing it is so much worse that she must scream, Nitrum.

**Cracking** in lumbar region extending to arms, Sul.

— in lumbar region when stooping, Agari.

— in lumbar region when walking, Zinc.

### LUMBAR REGION.

**Cramp**, jerk-like pain in lumbar region when sitting and lying, like a cramp, Bry.

**Cramp-like** sensation in left lumbar region, Bell.

— pain in lumbar region after long standing, when attempting to walk feels as if he would fall, Thuja.

— pain in lumbar region worse from least movement, *China.*

— pain in lumbar region with prolapsus uteri, *Bell.*

— pain in lumbo-sacral region, toward evening impeding every motion of the body at night and repeated the following evening, Sul.

**Crampy** pains in right lumbar region, Iris-v.

— pain and pinching in lumbar region and buttocks, *Caust.*

— pain at 5 P. M. in lumbar region, Diosc.

— pain in right loin in evening causing sweat, Iris-v.

— jerking pain from lumbar region to anus, *Calc.*

**Crick** in the back and lumbago, Calc-phos.

— in lumbar region a sudden "catch," *Sec.*

**Curvature** well marked in the lumbo-sacral region, *Sul.*

— of lumbar vertebræ, *Bell.*

— lumbar vertebræ bend forward with curvature of spine to left, *Calc-ph.*

**Cutting** in lumbar region hinders labor, *Kali-c.*

— as with glass in left lumbar region, Calad.

— deep pain in right loin transiently disappearing upon pressure. *Dulc.*

— and burning and scraping in lumbar region, Nat-c.

— in lumbar region so that he could not walk alone, Psor.

— violently in lumbar vertebrae, worse by stool, Rheum.

— and burning pains in lumbar region with sensativeness to touch, *Canth.*

— in lumbar region by sitting; afternoon extending to beneath shoulders where it became stitching, Canth.

— in lumbar region in cystitis, *Eup-pur.*

### LUMBAR REGION.

**Cutting** in lumbar region like an instrument going right through into the abdomen, Sul-ac.

— as from a knife through lumbar region, cannot walk, Kali-b.

— in lumbar region better by heat, *Sul.* (dry heat).

**Darting** in right lumbar region in A. M., Diosc., *Kali-iod.*

**Deep** seated pains in loins, with soreness from motion, Eup-per.

**Debility** great in lumbar region and profuse night sweats and symptoms of slow hectic fever, violent smarting and burning, *Phos-ac.*

**Diarrhœa,** painful soreness in lumbar region followed by frequent diarrhœa, *Bar-c.*

